# Prevalence of myopia and associated risk factors among key schools in Xi’an, China

**DOI:** 10.1186/s12886-022-02735-x

**Published:** 2022-12-30

**Authors:** Xingxing Zhao, Xin Lu, Lu Yu, Yiping Zhang, Jian Li, Yingyao Liu, Geqiang Yang, Yuan Wang, Wen Zhang, Zhaojiang Du

**Affiliations:** 1 Department of Ophthalmology, Xi’an Central Hospital 710005, Shaanxi Province, China; 2 Yan’an University, Yan’an City, Shaanxi Province, 716000 China

**Keywords:** Myopia, High myopia, Prevalence, Risk factors, Children, China

## Abstract

**Background:**

The aim of this study is to investigate the prevalence of myopia and high myopia and the associated risk factors in key schools in Xi'an, China.

**Methods:**

This cross-sectional study started in September 2021 and was conducted for one month. A total of 11,011 students from 10 key primary schools, five key junior high schools and five key high schools in Xi'an were randomly selected to undergo visual acuity measurement and non-cycloplegic autorefraction. The questionnaire was completed by the students and their parents together.

**Results:**

The prevalence of myopia and high myopia in key schools were 75.7% and 9.7%, respectively. The prevalence of myopia and high myopia rose significantly as grade or age increased (all *P* < 0.001), and the prevalence of myopia and high myopia in females was higher than that in males (*P* < 0.001, *P* < 0.5). According to the multivariable logistic regression analysis, older age (OR=1.42), female compared with male (OR=1.43), having one myopic parent (OR=1.64), having two myopic parent (OR=2.30) and often taking extracurricular tuition (OR=1.35) were more likely to be associated with develop myopia (*P* < 0.001). Older age (OR=1.39), having one myopic parent (OR=2.29), having two myopic parent (OR= 3.69), and often taking extracurricular tuition (OR=1.48) were more likely to be associated with high myopia (*P* < 0.001).

**Conclusions:**

The overall rate of myopia and high myopia in key schools in Xi'an, China, is extremely high. Myopia and high myopia are associated with increasing age, parents’ myopia, few outdoor exercises, and extracurricular tuition. Myopia is also associated with female and not having the habit of "one punch, one foot, one inch (when reading and writing, 10 cm from the chest to the table, 33 cm from the eye to the book and 3.3 cm from the tip of the pen to the finger)".

**Supplementary Information:**

The online version contains supplementary material available at 10.1186/s12886-022-02735-x.

## Background

Myopia has become the most the most common type of ametropia wordwide.Myopia,especially high myopia,is one of the main causes of visual impairment [1, 2] Holden et al [3] predicted that the number of people with myopia will reach 4.758 billion worldwide in 2050, or about 50% of the total population, and the number of people with high myopia will reach 938 million (about 9.8%). Myopia is characterized by a high incidence,early onset and rapid progression in East Asia.The rapid development of myopia, especially high myopia, needs to be considered because it not only leads to inconvenience in daily life but also causes ocular dieases,such as cataracts, glaucoma, retinal detachment and myopia macular degeneration [4–9].These conditions cause very large social and economic burdens [10, 11]. Therefore,myopia is of great concern,especially in East Asia.Currently, the large sample of epidemiological studies on myopia in Chinese children and adolescents has mostly focused on the myopia prevalence and its associated risk factors among randomly selected students. However, the prevalence of myopia and high myopia in some key schools with high academic burdens and good academic performance has not been determined. Therefore, it is necessary to analyse the latest prevalence and risk factors of myopia and high myopia in key schools in China to supplement the missing data of myopia epidemiological surveys, provide direction and theoretical basis for the prevention and control of myopia in children and adolescents and reduce the social and economic burden of the disease.

Therefore, we have assessed the prevalence of myopia and high myopia among children and adolescents in key schools in China and analysed the risk factors.

## Methods

### Study population

The current school-based cross-sectional study was conducted in Xi’an—a provincial capital city in East-Central China—from September 2021. We randomly selected a total of 11,011 students from 10 key primary schools, five key middle schools and five key high schools. Our survey of China’s education system is divided into three parts: primary school, junior high school and senior high school. Primary education lasts for six years, junior high for three years and senior high for three years. Each section is divided into regular schools and key schools. Compared with regular schools, the state and local governments concentrate and allocate limited human, material and financial resources to key schools. Key schools in China usually mean a richer learning environment, with better teacher resources, better electronic equipment resources, compared with regular schools.Students at key schools tend to spend more time on reading and completing learning tasks, while students from other school tend to have more outdoor time and exercise time [12].Students in key schools have a heavier academic burden and better academic performance than those in regular schools. All the students in the selected schools were examined for vision and refraction.The students wearing orthokeratology were defined as myopia population. The refractive error before wearing orthokeratology was adopted as the current refractive error of these students.Their parents and the students themselves filled out the questionnaires together. Those diagnosed with eye diseases such as leukoplakia, cataracts, glaucoma and retinal disease were excluded. The study followed the principles of the Declaration of Helsinki and was approved by the hospital’s ethics committee. Ophthalmology formally requests approval in writing, detailing the study objectives and procedures, and obtaining consent from the local education board to conduct the study in schools.

### Questionnaire and eye examinations

Using evidence from the literature [13–16], we designed a questionnaire to identify the associated risk factors of myopia and high myopia. To test the questionnaire, a total of 80 parents were randomly selected from primary, junior high and senior high schools, which were not included in the study. The parents were asked to provide feedback on how well the questionnaire was understood and how easy it was to fill out. Then, the questionnaire was determined according to the opinions of the parents.

The questionnaire included gender, date of birth, school grade, parental myopia, whether outdoor exercise regularly, regular exercise programme, daily sleep time, whether taking extracurricular tuition and whether doing ‘one punch, one foot, one inch’ (when reading and writing, 10 cm from the chest to the table, 33 cm from the eye to the book and 3.3 cm from the tip of the pen to the finger), whether eating green vegetables daily, whether eating sweets regularly, whether particular about food, and whether reading a book while travelling on public transport.

Visual acuity (VA) was assessed without refractive correction in all students, here by using a logarithmic VA chart with a 5-point recording at 5 m. Refractometry was performed in all students in a noncycloplegic state by autorefractometry (autorefractor KR-1, Topcon, Tokyo, Japan). The spherical equivalent (SE) refractive error was calculated as the sphere + 1/2 cylinder.

### Definitions

Since the refractive error was measured using non-cycloplegic autorefractor,which tended to over-measure the myopia magnitude,we defined myopia using combination of spherical equivalent and VA [17]. In our study, myopia is defined as VA>0.0 logMAR and spherical equivalent (SE)≤− 0.5 diopters (D) in at least one eye .High myopia was defined as VA>0.0 logMAR and spherical equivalent (SE)≤−6.0 diopters (D) in at least one eye.

### Statistical analysis

SPSS 26.0 was used for statistical analysis. To determine the associated risk factors of myopia and high myopia, univariable logistic regression analysis was used to calculate the odds ratios and 95% confidence intervals (Cl). Multivariable logistic regression analysis was used to determine the independent factors. All factors associated with myopia and high myopia identified in the univariable analysis were included in the multivariable analysis. Statistical tests were two-sided, and *P* < 0.05 was considered statistically significant.

## Results

### Subject characteristics

A total of 11,011 children finally took part in the study; here, 5,595 were boys, accounting for 50.8% of the study population, and 5,416 were female, accounting for 49.1% of the study population. The mean ± SD age was 13.48 ± 3.11 years old.

### Prevalence of myopia and high myopia

**Fig. 1:**
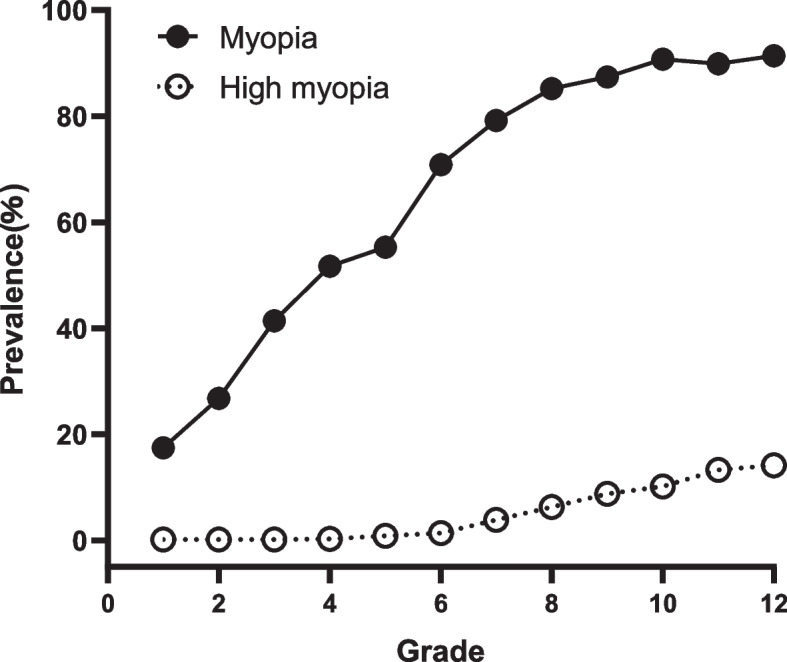
The prevalence rate of myopia and high myopia from grades 1 to 12

**Fig. 2:**
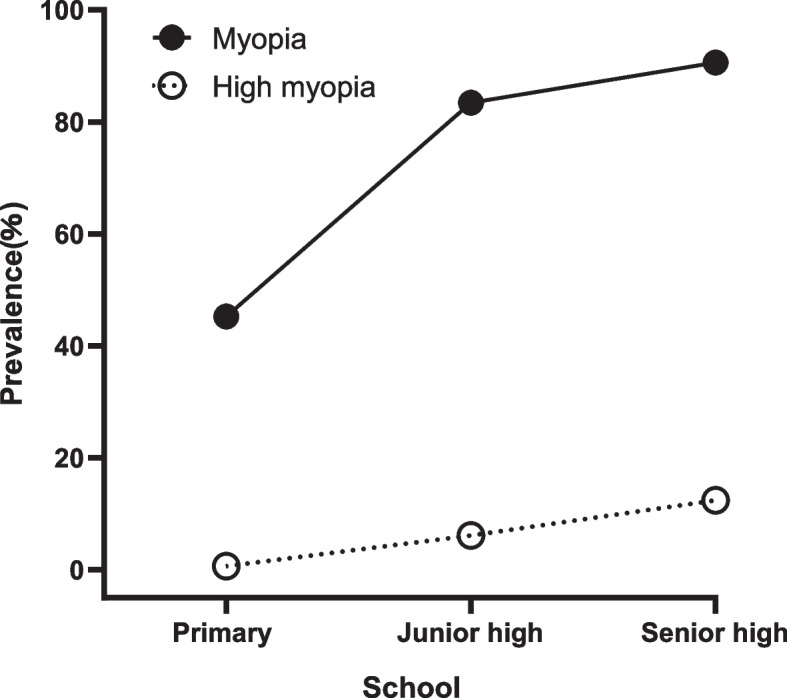
The prevalence rate of myopia and high myopia from different schools

The overall prevalence of myopia and high myopia in Xi’an’s key schools in 2021 were 75.7% and 9.7%, respectively. The prevalence of myopia and high myopia were 27.1% and 0.3% in patients under 7 years old and 86.1% and 10.2% in patients over 18 years old, respectively. The results showed that the prevalence of myopia and high myopia increased with age (all *P* < 0.001). Myopia and high myopia prevalence were 72.9% and 6.8% in boys and 78.5% and 7.8% in girls, respectively. The prevalence rates of myopia and high myopia in girls were higher than those in boys (*P* < 0.001, *P* < 0.5). The prevalence of myopia and high myopia were 45.2% and 0.6% for primary school students, 83.4% and 6.1% for junior high school students and 90.6% and 12.4% for senior high school students, indicating that the higher the school level, the higher the prevalence of myopia and high myopia (all *P* < 0.001) (Table 1). With the increase of grade level, the prevalence of myopia and high myopia increased (Fig. 1, Supplementary table 1). With the increase of school level, the prevalence of myopia and high myopia increased (Fig. 2).

**Table 1:** Prevalence of myopia and high myopia by student characteristics (*N* = 11011)

Demographics	N	Nonmyopia(-0.5<SE)	Myopia(SE≤-0.5)	High myopia (SE≤-6.0)
		n(%)	n(%)	n(%)
Age (years)			P<0.001	P<0.001
≤7^a^	642	468(72.9%)	174(27.1%)	2(0.3%)
8	501	323(64.5%)	178(35.5%)	3(0.6%)
9	505	294(58.2%)	211(41.8%)	1(0.2%)
10	444	227(51.5%)	217(48.9%)	1(0.2%)
11	596	254(42.6%)	342(57.4%)	6(1.0%)
12	902	249(27.6%)	653(73.4%)	36(4.0%)
13	1110	183(16.5%)	927(83.5%)	47(4.2%)
14	1084	157(14.5%)	927(85.5%)	83(7.7%)
15	1639	188(11.5%)	1451(88.5%)	142(8.7%)
16	1815	168(9.3%)	1647(90.5%)	246(13.6%)
17	1441	122(8.5%)	1319(91.5%)	204(14.2%)
≥18^b^	332	46(13.9%)	286(86.1%)	34(10.2%)
Gender			P<0.001	P<0.5
Male	5595	1515(27.1%)	4080(72.9%)	381(6.8%)
Female	5416	1164(21.5%)	4252(78.5%)	424(7.8%)
School			P<0.001	P<0.001
Primary school^c^	3159	1730(54.8%)	1429(45.2%)	18(0.6%)
Junior high school^d^	2978	493(16.6%)	2485(83.4%)	181(6.1%)
Senior high school^e^	4874	456(9.4%)	4418(90.6%)	606(12.4%)
Total	11011	2679(24.3%)	8332(75.7%)	805(9.7%)

^a^The minimum age was 6 years old

^b^The maximum age was 19 years old

^C^The age range is 6-14years old

^d^The age range is13-16 years old

^e^The age range is 15-19 years old

### Factors associated with myopia and high myopia

Univariable logistic regression analysis (Table 2) showed that age, gender, parental myopia, whether outdoor exercise regularly, regular exercise programme, daily sleep time, whether taking extracurricular tuition, whether doing ‘one punch, one foot, one inch’, whether eating green vegetables daily, whether eating sweets regularly, and whether particular about food were associated with myopia (*P* < 0.05). Multivariable logistic regression analysis of the above factors (Table 3) showed that students who were older (OR=1.42), female compared with male (OR=1.43), having one myopic parent (OR=1.64), having two myopic parent (OR=2.30) and often taking extracurricular tuition (OR=1.35) were more likely to be associated with develop myopia (*P* < 0.001) . Conducting outdoor exercise regularly (OR=0.79) and doing ‘one punch, one foot, one inch’ (0R=0.81) reduced the risk of myopia (*P* < 0.001); students who were older (OR=1.39), having one myopic parent (OR=2.29), having two myopic parent (OR= 3.69), and often taking extracurricular tuition (OR=1.48) were more likely to be associated with high myopia (*p* < 0.001). Conducting outdoor exercise regularly (OR=0.77) reduced the risk of high myopia (*P* < 0.05).

**Table 2:** Single variable logistic regression analysis of the risk factors associated with myopia and high myopia

		Myopia			High myopia	
	Beta	OR(95% CI)	P	Beta	OR(95% CI)	P
Age, y	0.36	1.43(1.40–1.45)	<0.001	0.33	1.40(1.35–1.45)	<0.001
Gender						
Male	Reference	Reference		Reference	Reference	
Female	0.31	1.36(1.24–1.48)	<0.001	0.15	1.16(1.01–1.34)	=0.04
Parental myopia						
No	Reference	Reference		Reference	Reference	
Father or Mother	0.40	1.49(1.34–1.65)	<0.001	0.80	2.23(1.91–2.61)	<0.001
Both	0.79	2.20(1.72–2.82)	<0.001	1.28	3.60(2.81–4.61)	<0.001
Whether outdoor exercise regularly						
No	Reference	Reference		Reference	Reference	
Yes	-0.61	0.54(0.49–0.59)	<0.001	-0.55	0.58(0.50–0.67)	<0.001
Regular exercise programme						
Other	Reference	Reference		Reference	Reference	
Basketball	0.09	1.09(0.96–1.24)	=0.175	-0.09	0.92(0.75–1.13)	=0.425
Table tennis	0.10	1.10(0.94–1.28)	=0.226	-0.08	0.92(0.72–1.19)	=0.528
Badminton	-0.01	0.99(0.89–1.11)	=0.903	0.00	1.00(0.84–1.20)	=0.992
Football	-0.48	0.62(0.52–0.74)	<0.001	-0.65	0.53(0.36–0.77)	=0.001
Daily sleep time						
<8 hours	Reference	Reference		Reference	Reference	
≥8 hours	-0.60	0.55(0.50–0.60)	<0.001	-0.61	0.55(0.47–0.64)	<0.001
Whether taking extracurricular tuition						
No	Reference	Reference		Reference	Reference	
Yes	0.54	1.71(1.51–1.94)	<0.001	0.55	1.74(1.48–2.04)	<0.001
		Myopia			High myopia	
	Beta	OR(95% CI)	P	Beta	OR(95% CI)	P
Whether doing "one punch, one foot, one inch "						
No	Reference	Reference		Reference	Reference	
Yes	-0.65	0.52(0.48–0.57)	<0.001	-0.44	0.65(0.56–0.75)	<0.001
Whether eat green vegetables daily						
No	Reference	Reference		Reference	Reference	
Yes	-0.24	0.79(0.69–0.90)	=0.001	-0.05	0.96(0.77–1.18)	=0.677
Whether eating sweets regularly						
No	Reference	Reference		Reference	Reference	
Yes	0.25	1.29(1.17–1.42)	<0.001	0.07	1.07(0.92–1.24)	=0.406
Whether particular about food						
No	Reference	Reference		Reference	Reference	
Yes	0.17	1.18(1.07–1.30)	=0.001	0.09	1.10(0.94–1.28)	=0.257
Whether reading a book while travelling on public transport						
No	Reference	Reference		Reference	Reference	
Yes	0.13	1.14(0.94–1.38)	=0.191	0.22	1.24(0.93–1.65)	=0.137

**Table 3:** Multivariable logistic regression analysis of the risk factors associated with myopia and high myopia

		Myopia			High myopia	
	Beta	OR(95% CI)	P	Beta	OR(95% CI)	P
Age, y	0.35	1.42(1.39–1.44)	<0.001	0.33	1.39(1.34–1.45)	<0.001
Gender						
Male	Reference	Reference		Reference	Reference	
Female	0.36	1.43(1.29–1.59)	<0.001	0.05	1.05(0.89–1.24)	=0.54
Parental myopia						
No	Reference	Reference		Reference	Reference	
Father or Mother	0.50	1.64(1.46–1.85)	<0.001	0.83	2.29(1.95–2.69)	<0.001
Both	0.83	2.30(1.74–3.03)	<0.001	1.31	3.69(2.84–4.79)	<0.001
Whether outdoor exercise regularly						
No	Reference	Reference		Reference	Reference	
Yes	-0.24	0.79(0.70–0.88)	<0.001	-0.27	0.77(0.65–0.90)	=0.001
Regular exercise programme						
Other	Reference	Reference		Reference	Reference	
Basketball	-0.07	0.94(0.80–1.09)	=0.401	-0.11	0.89(0.71–1.12)	=0.327
Table tennis	-0.17	0.84(0.71–1.01)	=0.059	-0.17	0.84(0.65–1.10)	=0.205
Badminton	-0.19	0.83(0.73–0.95)	=0.005	-0.08	0.93(0.77–1.12)	=0.419
Football	-0.18	0.83(0.68–1.02)	=0.077	-0.35	0.70(0.47–1.05)	=0.084
Daily sleep time						
<8 hours	Reference	Reference		Reference	Reference	
≥8 hours	-0.09	0.92(0.83–1.02)	=0.103	-0.12	0.89(0.75–1.05)	=0.158
Whether taking extracurricular tuition						
No	Reference	Reference		Reference	Reference	
Yes	0.30	1.35(1.18–1.56)	<0.001	0.39	1.48(1.24–1.75)	<0.001
		Myopia			High myopia	
	Beta	OR(95% CI)	P	Beta	OR(95% CI)	P
Whether doing "one punch, one foot, one inch "						
No	Reference	Reference		Reference	Reference	
Yes	-0.21	0.81(0.73–0.91)	<0.001	-0.10	0.90(0.77–1.06)	=0.203
Whether eat green vegetables daily						
No	Reference	Reference		Reference	Reference	
Yes	0.02	1.02(0.86–1.20)	=0.857	0.18	1.20(0.95–1.51)	=0.124
Whether eating sweets regularly						
No	Reference	Reference		Reference	Reference	
Yes	0.48	1.05(0.94–1.18)	=0.404	-0.11	0.90(0.76–1.06)	=0.204
Whether particular about food						
No	Reference	Reference		Reference	Reference	
Yes	-0.09	0.92(0.81–1.03)	=0.156	-0.05	0.95(0.80–1.13)	=0.558

## Discussion

ThE current study was conducted in a key school in Xi’an, Shaanxi Province, China, and found that the overall myopia prevalence among children and adolescents aged 6–19 years was 75.7% and high myopia prevalence 9.7%, with the myopia rate in grade 1 being 17.5% and high myopia rate being 0.2%, and by grade 12 the myopia rate was as high as 91.4% and high myopia rate was 14.2%.which was higher than the myopia prevalence of children and adolescents in Wenzhou, Beijing and Chongqing [12, 13, 18]. It is also higher than the rate of myopia announced by the World Health Organization in 2020 (35.6% in primary school, 71.1% in middle school and 80.5% in high school).

Consistent with the relevant literature at home and abroad [19], the present study has shown that the prevalence of myopia and high myopia of females was higher than that of males. It is speculated that females prefer read books during recess, which increases the time for near work and decreases the time for outdoor exercise, which may be linked to increased myopia in females [20]. In 2015, Ye et al [21] reported that rs9307551 gene associated with high myopia exists in Han female population, which also confirmed that the high myopia rate in girls is higher than that in boys.Other studies [22] have shown that the higher myopia prevalence of females than males is associated with the level of sex hormones in their bodies, but further studies are needed to confirm whether high myopia is associated with sex hormones. The results of the current study showed that the myopia prevalence increased with the learning stage and age, that is, senior high school > junior high school > primary school. Many previous studies have also shown that myopia is closely associated with grade [23, 24], which may be associated with the increase of school level, increased workload and reduced time for outdoor exercise, resulting in excessive use of the eye and continuous high tension of the ciliary and extraocular muscles. The present study found that myopia prevalence increased rapidly in primary school, especially in the lower grade group, which should be regarded as a key target for prevention and control. The prevention and control of myopia should start in the lower grade of primary school [25].

Logistic regression analysis showed that older age, parental myopia, less frequent outdoor exercise and extracurricular tuition were associated with an increase in the prevalence of myopia and high myopia. Myopia also associated with gender and not having the habit of "one punch, one foot, one inch ", which was consistent with relevant research results at home and abroad [14, 26–28]. Taking extracurricular tuition and not having the habit of "one punch, one foot, one inch " were risk factors for working in close proximity for a long time. A longitudinal survey also showed that proper rest after a short period of short distance (> 30 cm) work can continuously reduce the prevalence of myopia in children. Increased durations of near work can easily cause asthenopia, accommodative lag and a defocused state, which can induce compensatory axial growth and promote the occurrence and development of myopia. By investigating these risk factors for myopia and high myopia, we can better identify those who may need an intervention to reduce the development of high myopia in those students who have already become myopia. It can also help in understanding the pathophysiological mechanisms of the occurrence and development of myopia.

It was discovered that risk of myopia and high myopia increased with a positive family history, in agreement with the viewpoint that familial effects on the level and onset of myopia [29],and genetic factors play importantly in myopic development [30].The current study found that frequent outdoor exercise is a protective factor against myopia and high myopia. The results of a recent prospective study suggest that prolonged exposure to high-intensity sunlight can slow axial eye growth [31]; high-intensity sunlight may be the reason why outdoor exercise prevents the progression of myopia [32]. Basic studies have shown that when retinal neuronal activity is enhanced, the activation of tyrosine hydroxylation maintains the steady reserve of dopamine, and rapid changes in outdoor light and brightness with high intensity may stimulate the synthesis and release of dopamine [33], thus alleviating the growth of myopia. Currently, there are different opinions about the effective duration of outdoor exercise. A clinical trial of increasing outdoor exercise time in children and adolescents to reduce the occurrence and development of myopia may be more beneficial for quantifying the relationship between light exposure intensity and activity duration and occurrence and development of myopia.

We conducted a study on the latest prevalence and risk factors for myopia and high myopia in key schools in China. One advantage of the current study is that we selected a large sample size of students at all grade levels, allowing us to assess the relationship between myopia and high myopia and age, grade level and gender. However, there are some limitations. First, the refraction of each student was assessed by non-cycloplegic autorefraction, which could result in an overestimation of myopia prevalence.We tried to reduce the overestimated prevalence by basing the definitions of myopia on the combination of SE and vision. This should reduce the number of emmetropes falsely classified as (mildly) myopic.Nevertheless, our estimates of myopia and high myopia are still biased.Second,our research lacks research on non-key schools in this city, and only indirectly shows that the rate of myopia in key schools in this city is higher by comparing the rate of myopia in other regions of China.Third, most risk factors were obtained by filling out questionnaires, so recall bias is inevitable.

## Conclusion

This is a cross-sectional study based on key school discovery. The overall prevalence of myopia and high myopia in key schools in Xi'an, China, is very high. The higher the grade or age, the higher the prevalence of myopia and high myopia; for example, the prevalence of myopia in senior high school students reached 90%. Currently myopia has become a serious public health problem closely associated with the heavy academic burden of students. Myopia and high myopia are associated with increased age, parental myopia, less outdoor exercise and extracurricular tuition. Myopia also associated with female and not having the habit of "one punch, one foot, one inch ".

## Supplementary Information

**Additional file 1.**

**Additional file 2.**

## Data Availability

The detasets generated during and analysed during the current study are not available due to the protection of data security (the original data contains a lot of specifically demographic characteristics information and will be used again in the future folloe-up study) but are available from the corresponding author on reasonable request.
