# Supplementary material for: Prevalence of myopia and associated risk factors among key schools in Xi’an, China
Source: BMC Ophthalmol. 2022 Dec 30;22:519. doi: 10.1186/s12886-022-02735-x (PMC9801531; doi:10.1186/s12886-022-02735-x)
Supplement: Supplementary file 1 — Additional file 1. [file 12886_2022_2735_MOESM1_ESM.docx]

**Supplementary table 1.** Prevalence of myopia and high myopia by characteristics of students (N = 11011)

| Demographics  (age range in years) | N | Non-myopia(-0.5＜SE) | Myopia(SE≤-0.5) | High myopia (SE≤-6.0) |
| --- | --- | --- | --- | --- |
|  |  | n(%) | n(%) | n(%) |
| Grade |  |  | P＜0.001 | P＜0.001 |
| Grade1(6-9) | 474 | 391(82.5%) | 83(17.5%) | 1(0.2%) |
| Grade2(8-10) | 512 | 375(73.2%) | 137(26.8%) | 1(0.2%) |
| Grade3(9-11) | 466 | 273(58.6%) | 193(41.4%) | 1(0.2%) |
| Grade4(10-12) | 536 | 259(48.3%) | 277(51.7%) | 2(0.3%) |
| Grade5(11-13) | 584 | 261(44.7%) | 323(55.3%) | 5(0.9%) |
| Grade6(12-14) | 587 | 171(29.1%) | 416(70.9%) | 8(1.4%) |
| Grade7(13-14) | 1181 | 246(20.8%) | 935(79.2%) | 46(3.9%) |
| Grade8(14-16) | 932 | 138(14.8%) | 794(85.2%) | 59(6.3%) |
| Grade9(15-16) | 865 | 109(12.6%) | 756(87.4%) | 76(8.8%) |
| Grade10(15-17) | 1786 | 165(9.2%) | 1621(90.8%) | 183(10.2%) |
| Grade11(16-18) | 1680 | 170(10.1%) | 1510(89.9%) | 223(13.3%) |
| Grade12(17-19) | 1408 | 121(8.6%) | 1287(91.4%) | 200(14.2%) |
| Total(6-19) | 11011 | 2679(24.3%) | 8332(75.7%) | 805(9.7%) |
