# Supplementary material for: Prevalence of myopia and associated risk factors among key schools in Xi’an, China
Source: BMC Ophthalmol. 2022 Dec 30;22:519. doi: 10.1186/s12886-022-02735-x (PMC9801531; doi:10.1186/s12886-022-02735-x)
Supplement: Supplementary file 2 — Additional file 2. [file 12886_2022_2735_MOESM2_ESM.docx]

**Eye survey questionnaire**

School：___________________ Name：___________________

Age ：___________________ ID：_____________________

## Class and grade：___________________

1. Gender

□ male □ female

1. Is your father nearsighted?

□ Yes □ No

1. Is your mather nearsighted?

□ Yes □ No

1. Have you worn orthokeratology lens in the last month?

□ Yes □ No

If yes, the diopter of the right eye wearing a orthokeratology lens is___________

If yes, the diopter of the left eye wearing a orthokeratology lens is___________

1. Whether outdoor exercise regularly? \

□ Yes □ No

1. Regular exercise programme

□ Basketball □Table tennis □Badminton □Football □ Other

1. Daily sleep time

□ ＜8 hours □ ≥8 hours

1. Whether taking extracurricular tuition?

□ Yes □ No

1. Whether doing "one punch, one foot, one inch (when reading and writing, one punch from the chest to the table, 33 cm from the eye to the book and 3.3 cm from the tip of the pen to the finger) "?

□ Yes □ No

1. Whether eat green vegetables daily?

□ Yes □ No

1. Whether eating sweets regularly?

□ Yes □ No

1. Whether particular about food?

□ Yes □ No

1. Whether reading a book while travelling on public transport?

□ Yes □ No
